# Fidelity, Feasibility and Adaptation of a Family Planning Intervention for Young Women in Zimbabwe: Provider Perspectives and Experiences

**DOI:** 10.1007/s43477-023-00075-6

**Published:** 2023-03-24

**Authors:** Constancia V. Mavodza, Sarah Bernays, Constance R. S. Mackworth-Young, Rangarirayi Nyamwanza, Portia Nzombe, Ethel Dauya, Chido Dziva Chikwari, Mandikudza Tembo, Tsitsi Apollo, Owen Mugurungi, Bernard Madzima, Dadirai Nguwo, Rashida Abbas Ferrand, Joanna Busza

**Affiliations:** 1 The Health Research Unit Zimbabwe, Biomedical Research and Training Institute, 10 Seagrave Road, Avondale, Harare, Zimbabwe; 2 Department of Public Health, Environments and Society, Faculty of Public Health and Policy, London School of Hygiene and Tropical Medicine, London, UK; 3 Department of Global Health and Development, Faculty of Public Health and Policy, London School of Hygiene and Tropical Medicine, London, UK; 4 School of Public Health, University of Sydney, Sydney, Australia; 5 Department of Infectious Diseases Epidemiology, Faculty of Epidemiology and Population Health, London School of Hygiene and Tropical Medicine, London, UK; 6 HIV and TB Department, Ministry of Health and Child Care, Harare, Zimbabwe; 7 National AIDS Council, Harare, Zimbabwe; 8 Population Services Zimbabwe, Harare, Zimbabwe; 9 Department of Clinical Research, Faculty of Infectious and Tropical Diseases, London School of Hygiene and Tropical Medicine, London, UK

**Keywords:** Implementation fidelity, Adaptation, Feasibility, Quality, Family planning services, Zimbabwe

## Abstract

The CHIEDZA (Community-based Interventions to improve HIV outcomes in youth: a cluster randomised trial in Zimbabwe) trial evaluated an integrated package of HIV and sexual and reproductive health services for young people aged 16–24 years in Zimbabwe. The family planning component aimed to improve access to information, services, and contraceptives delivered by trained youth-friendly providers within a community-based setting for young women. Responsively adapting the intervention was a part of the intervention design’s rationale. We investigated the factors influencing implementation fidelity, quality, and feasibility using provider experiences and perspectives. We conducted provider interviews (*N* = 42), non-participant (*N* = 18), and participant observation (*N* = 30) of intervention activities. The data was analyzed thematically. CHIEDZA providers were receptive to providing the family planning intervention, but contexts outside of the intervention created challenges to the intervention’s fidelity. Strategic adaptations were required to ensure service quality within a youth-friendly context. These adaptations strengthened service delivery but also resulted in longer wait times, more frequent visits, and variability of Long-Acting Reversible contraceptives (LARCS) provision which depended on target-driven programming by partner organization. This study was a practical example of how tracking adaptations is vital within process evaluation methods in implementation science. Anticipating that changes will occur is a necessary pre-condition of strong evaluations and tracking adaptations ensures that lessons on feasibility of design, contextual factors, and health system factors are responded to during implementation and can improve quality. Some contextual factors are unpredictable, and implementation should be viewed as a dynamic process where responsive adaptations are necessary, and fidelity is not static.

*Trial registration* ClinicalTrials.gov Identifier: NCT03719521.

**Supplementary Information:**

The online version contains supplementary material available at 10.1007/s43477-023-00075-6.

Barriers to young people’s access to sexual and reproductive health (SRH) services in sub-Saharan Africa have been well documented and remains a challenge (Denno et al., 2015; Kennedy et al., 2010; Phillips & Mbizvo, 2016). Despite numerous interventions to address family planning for young women, utilization remains persistently low (MacQuarrie, 2014; Mutumba et al., 2018).

In Zimbabwe, the unmet need for family planning among young unmarried sexually active women is 37% (15–19 years) and 17% (20–24 years), compared to the national unmet need of 12.6% (Zimbabwe National Statistics Agency & International., 2016). Negative attitudes and judgmental health providers dissuade young people from seeking SRH care (Amnesty International, 2018). Despite the Ministry of Health and Child Care (MoHCC)’s prioritization of adolescent SRH in key planning documents, youth-friendly integrated SRH services remain largely inaccessible for young people (MoHCC, 2016a, 2016b), and are offered vertically with limited integration (Church & Mayhew, 2009; Warren et al., 2017).

CHIEDZA is a pragmatic cluster-randomized trial investigating the impact of community-based integrated HIV and SRH services for young people aged 16–24 years on population-level HIV outcomes. The trial was conducted in three provinces in Zimbabwe: Harare, Bulawayo, and Mashonaland East (Dziva Chikwari, 2022) with each province having four intervention and four control clusters. The intervention was implemented for 30 months in community centers in each intervention cluster by a team that included nurses, community health workers, counsellors, and youth workers. CHIEDZA was conceived based on the rationale that offering youth-friendly, integrated (one-stop-shop) HIV and SRH services outside of facility settings (community-setting), would increase engagement with, and uptake of services by youth (Dziva Chikwari, 2022). One-stop-shop services are ones where all the services are provided under the same roof, ideally by the same providers (Wood & Aggleton, 2003). Family planning is one component of the CHIEDZA trial intervention. It was anticipated from the outset that adaptations would be required during the trial to respond to contextual issues across settings (Barratt et al., 2016). As part of the trial, a process evaluation was conducted alongside delivery of the intervention as a recognized method to understand realities of implementation, including whether, how and why the intervention operates as anticipated (Moore et al., 2015).

Process evaluations have become an integral element of evaluating complex interventions that are multi-component and context-dependent (Moore et al., 2015). They seek to understand what is working, for whom, when, and how (Oakley et al., 2006). Process evaluation concepts have existed since the 1960s and have developed and diversified over time and some of the key ones like fidelity, are also concepts in implementation research broadly (Quasdorf et al., 2021; Rabin et al., 2008). Historically, fidelity has been viewed as commitment to the intervention as it was originally intended by its designers (Collins et al., 2004; Quasdorf et al., 2021). However, considerations for contextual influences have substantiated the potential co-existence of fidelity and adaptations (Cannata et al., 2021; Moore et al., 2021; Pérez et al., 2016). One of process evaluations and implementation research’s intended aims can be to anticipate and enable such adaptations (Greenhalgh et al., 2004; Moore et al., 2021).

During interventions, frontline providers play a critical role in determining the intervention’s feasibility due to their willingness to deliver planned activities (Amoakoh et al., 2019; Sekhon et al., 2017). Providers’ readiness to accept the intervention is a determinant of feasibility where their skill and motivations are key to devising and ensuring that an intervention is acceptable to its recipients (Sekhon et al., 2017). Therefore, providers are critical informants for capturing the implementation processes of an intervention (Damschroder et al., 2009). This paper examines the perceptions, experiences, and opinions of the frontline providers in CHIEDZA, and is part of the broader process evaluation*.*

The aim of this study was to assess and understand the complexities of implementing a family planning services, and the responsive adaptive processes that were adopted. To do this, we sought to: (1) assess whether the family planning intervention within CHIEDZA was implemented as intended (fidelity); (2) understand the feasibility of implementing the intervention; (3) assess quality of the intervention and identify unintended consequences; and (4) examine the contextual underpinnings of fidelity, feasibility, and quality.

## Family Planning in CHIEDZA

**Fig. 1:**
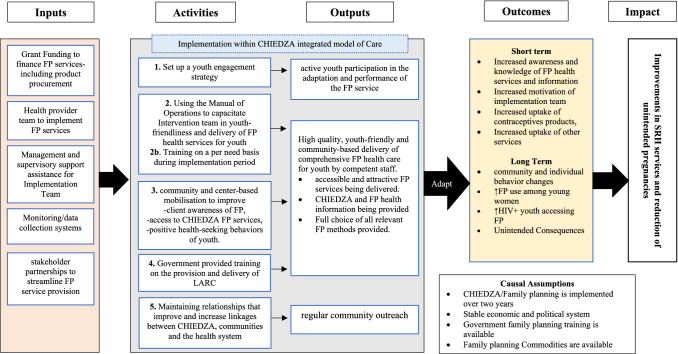
Model describing the implementation of the family planning intervention as intended, including the anticipated outcomes and impact

The family planning service delivery model (Fig. 1) in CHIEDZA aimed to contribute to the MoHCC’s goal of providing accessible family planning services to young people, with a focus on provision of long-acting reversible contraceptives to increase access to and use of family planning by young people (MoHCC, 2016a). The delivery model aligned with the MoHCC’s systems through procuring commodities through the government’s supply chain and reporting family planning uptake to Ministry’s registers. In Zimbabwe, Secure is the brand name of the progesterone-only contraceptive pill, Control is the brand name for combined oral contraceptive pills, Jadelle is a brand name for an implant and Depo refers to the Depo Provera injectable. To become a family planning nurse, qualified nurses must undergo government-run family planning specific training. While most nurses can offer oral contraceptives, long-acting reversible contraceptives (implants and Intra-uterine contraceptive devices) can only be provided by nurses with additional training from the government program.

Young women attending CHIEDZA could hear about the available family planning services from youth community mobilisers, the youth workers at CHIEDZA or the service providers at the CHIEDZA sites. Every young person who entered the health booth was offered HIV testing and treatment if applicable, and risk reduction counselling as well as condoms, management of sexually transmitted infections, referral for voluntary male medical circumcision (for males), and health counselling services, and menstrual health education, and products (for females). Female clients were offered family planning services including information, education, and counselling, as well as oral contraceptives (combined oral contraceptives and progesterone-only contraceptives), Depo, emergency contraceptives, and pregnancy testing. First time users of oral contraceptives were initially given a one-month supply to enable monitoring of side effects. Thereafter, three monthly supplies were given. The long-acting reversible contraceptives were provided by a non-governmental organization, Population Services Zimbabwe (PSZ), which employed government-trained nurses.

When PSZ was located at CHIEDZA centers, they also offered services to older women (over 24 years), and therefore they had to operate in a separate booth to avoid diluting the focus on youth. The nurse provided the first consultation, counselling, and information on all family planning products and services offered at CHIEDZA and PSZ. Clients then selected and were provided with their contraceptive or service of choice. At subsequent visits, they could receive contraceptive pill refills from community health workers. Those who used injectables and experienced any complications/side effects were always served by nurses. Outside of the CHIEDZA community centers, information on the available family planning services were also offered via community mobilization efforts by youth champions, which included flyer distribution and peer-to-peer interactions.

## Methods

### Study Design: Process Evaluation

We used the Medical Research Council’s process evaluations framework that looks at implementation, mechanisms of change, and context of complex interventions (Moore et al., 2015). To describe and understand implementation and adaptation processes we focused on three evaluation outcomes: (1) fidelity (2) feasibility, and (3) quality of the service being delivered. Fidelity refers to the extent to which an intervention is delivered as intended (Moore et al., 2015). In the case of CHIEDZA, adaptation was included as an ongoing part of the intervention design (Fig. 1). As part of the family planning intervention, adaptations could be made in response to need and context (responsive adaptations). To enable these adaptations, the CHIEDZA teams (research team and implementing teams) met every month to discuss progress, challenges, and opportunities within the intervention. It was during these meetings that decisions to adapt where made, based on not only reports and service provision experiences of the providers, but also based on feedback from the process evaluation team if they had interviewed youth clients and/or the providers. These regular team meetings were in place for both decision-making and documentation of agreed changes. Thus, in our process evaluation, fidelity refers to the adapted implementation model as re-designed over time. Feasibility is often examined as part of pilot studies, to determine to what extent a departure from fidelity is due to supply side challenges or gaps (McLeod, 2021). While “feasibility studies” sometimes refer to pilot interventions, the viability of provision remains relevant throughout delivery of any intervention, necessitating feasibility as a key component of a process evaluation. Quality refers to whether providers were able to provide youth-friendly, non-judgmental, time efficient, integrated, family planning services with adequate choice of contraceptives. These components may directly or indirectly affect each other and subsequent outcomes.

### Eligibility and Participant Recruitment

Health providers from the three provinces were eligible to participate in the study. Purposive sampling ensured that there was representation of each cadre of health provider (community health worker, nurse, counsellor, and youth worker). All invited providers agreed to participate. At each phase of data collection all cadres of providers were interviewed and repeat interviews with at least one nurse, community health worker, youth worker, and counsellor were conducted to gauge changing perspectives over time.

### Data Collection

Interview topic guides and observation guides were the main data collection tools. The main qualitative researcher (CM) was located with the CHIEDZA trial since its inception, and the CHIEDZA providers interacted with her frequently, leading to an established rapport.

#### Provider Interviews

Between April 2020–November 2021, 42 interviews with 27 CHIEDZA providers were conducted. Data were collected at three time points (Table 1). These time points ensured that the process of adaptation and related adjustments, implementation, and feasibility over time, as well as dramatic contextual shifts (COVID-19) in intervention delivery, could be captured. Each data collection phase built upon the previous one through an iterative process (Table 1). Topic guides had open-ended questions. The phase 1 interviews (*n* = 16) sought to understand, very broadly, the family planning issues or concerns that young people raised with providers at CHIEDZA. The phase 2 (*n* = 15) interviews explored providers’ perceptions of the service delivery model and its implementation. The phase three interviews (*n* = 11) were conducted at month 25/30, when the CHIEDZA intervention was ending and focused on providers’ reflection on family planning services over time (Table 1). All interviews were audio-recorded.

**Table 1:** Qualitative data collection timelines, participants, methods, and areas of exploration

Phase	Sampling strategy	Type of interview participants	Data collection method	Area of exploration
1. Apr 2020	Purposive sample: each province and type of health provider represented	16 health providers (10 females; 6 males)	Phone interviews	Experiences of implementing Family planning, including early COVID-19 effects; perceptions of training received, and service delivery model employed
2. Jul–Aug 2020	Purposive sample: each province and type of health provider represented; repeat interviews for change over time	15 health providers (10 females; 5 males) 7 repeats; 8 new interviews)	5 Phone interviews 10 In-person interviews	Experiences of implementing family planning & perceptions of service delivery model employed; challenging family planning issues/ concerns that young people raise and how providers meet these challenges
3. Oct–Nov 2021	Purposive sample: each province and type of healthcare provider represented; repeat interviews for change over time	11 health providers (6 females; 5 males) 6 repeats; 5 new interviews)	In-person interviews	The implementation of the trial is tapering to an end at this point Experiences of implementing family planning services at CHIEDZA over time, what worked or didn’t work overtime?
Apr 2019–Dec 2021	All	Debrief meetings Participant Observations	Meeting minutes and field notes	Real time family planning service provision experiences of health providers Adaptation decisions made during or as part of these meetings
June 2020–July 2021	Purposive sample: each province; clusters where PSZ was present	18 non-participant observation events	Observation forms and field notes	Contextual understanding of implementing family planning services at the community centers

All phase 1 interviews occurred during the first week of COVID-19 lockdown and were telephonic. For these, written consent was obtained during the last in-person team meeting two days before interviews began. The lead researcher (CM) contacted those who had consented to arrange telephonic interviews. Twenty providers consented and 16 were interviewed: two did not respond to contact attempts, one was an oversampled cadre (community health worker), and one was excluded based on their temporary position on the team. Phase 2 interviews were conducted during a less severe lockdown that still restricted intercity travel, so interviews were either in-person or telephonic. With the intercity telephone interviews, a local research assistant obtained in-person, written consent from the providers in the province to which we could not travel. CM then conducted the interviews telephonically as in phase one. The in-person interviews were conducted by CM and RN after written consent had been obtained. Phase 3 interviews were conducted in-person by RN and PN, during moderate lockdown measures, with providers who gave written consent. All in-person interviews took place in private rooms at the research offices on days that the providers did not have to be at the community centers providing services.

All interviews were conducted in either English, Shona, or Ndebele, depending on participants’ preference, and transcribed into English. Each interview took between 30 and 100 min. All transcripts were anonymized to maintain confidentiality.

#### Non-participant Observations at CHIEDZA Community Centers

The purpose of visits to CHIEDZA centers was to observe how providers implemented family planning services, including interactions with provision of other SRH services. The researchers did not participate in service provision. Observations enable the examination of contextual influences (Tashakkori & Teddlie, 2010), and we assessed interactions between providers, community members, youth clients, and the family planning service. Field notes, guided by an observation guide, were written immediately in real-time. They were then reviewed and written up into a more detailed description 24–48 h later (Walford, 2009; Wolfinger, 2002).

#### Participant Observations and Meeting Minutes

Participant observations and minutes of meetings between the research and implementation (CHIEDZA providers and coordinators) teams (N = 30) occurred between April 2019–December 2021. CM or RN attended meetings as both a participant contributing to the team discussions (participant role), and as a researcher observing and taking notes and minutes. The observations and meeting minutes captured real-time experiences and perspectives of providing family planning services, including any decisions made by the broader team to adapt/change the implementation model for improvement.

### Data Analysis

CM familiarized herself with 42 transcripts, 30 meeting documents, and 18 observation summaries. Thematic analysis was employed, and initially descriptive codes were arranged into emerging inductive themes, compiled in data summary notes. Summaries were further developed into analytical memos exploring connections between phases, highlighting significant themes, and distilling ideas that materialized (Birks et al., 2008). Themes included perceptions of the family planning service delivery model, barriers, facilitators, and recommendations for implementation; client flow for family planning; service provider roles and responsibilities; commodity availability; and fidelity to the implementation strategy and intervention. The analytical processes were iterative and occurred as data were being collected with phase-to-phase comparison of emerging themes. The collaborative analytical process involved discussions amongst CM, SB, and JB.

## Results

### Implementing the Family Planning Service Delivery Model

According to the providers, the CHIEDZA family planning service delivery model was based on the hypothesis that if provided with adequate information, young women could make an informed decision on their contraceptive method of choice. Information on family planning methods was provided to clients at various points of the CHIEDZA client flow, from the youth workers at first point of contact who gave health education talks, then the community health worker in the health booth, and then the nurse who prescribed the method. Across the different points, this information varied in-depth based on the expertise of the provider. The providers considered the intervention to be broadly appropriate, and they clarified that they spent time and effort informing clients about each contraceptive on offer, and the side effects so that clients could make informed contraceptive decisions

When a client came, they would see the youth worker and we would talk about what we offer at the social area first then when they got into the booth that’s when they would tell the CHW [community health worker] which family planning method they wanted personally. The CHW will also tell the client the methods available that very day if PSZ wasn’t there. So, the methods we usually offered when PSZ wasn’t there were the Control, Secure, and Depo and then the client would choose what method they wanted…the CHW would properly explain that if they want more detail on the methods, they will get it in the nurses’ booth...When they come to my tent, I would ask them what they know about the method they have chosen... When you explained the side effects [Depo for example] that’s when they would switch to another method... (Harare, IDI02, Phase3)

Providers noted that they also invited clients to ask questions about family planning or contraceptives. In some instances, providers were asked to verify or deny information about contraceptives that clients heard. Sometimes, providers felt that they did not have adequate responses or answers to these questions, which for them, compromised their ability to provide quality information to young women. However, over time, experiential learning improved their knowledge of family planning and contraceptives.

It [knowledge of family planning] has improved so much! It has! Now I am partly responsible in helping clients choose their family planning service that they may think is suitable for them (Bulawayo, IDI04, Phase 2)

### Factors Affecting Implementation

Implementing the family planning intervention was affected by significant factors and events that influenced where, when, and how young women received, and providers delivered the intervention (Supplementary Table 1). Affected intervention components included contextual events in Zimbabwe, and partnerships within the intervention. Contextual events refer to those that occurred in the country during the implementation period and influenced how the intervention was implemented and experienced by CHIEDZA providers.

#### National Contraceptive Shortage

In September 2019, a national-level shortage of all contraceptive commodities was announced. At this point, CHIEDZA was procuring contraceptives from private suppliers (Supplementary Table 1), and the availability of family planning commodities at CHIEDZA was affected. In the lead up to the announcement, CHIEDZA providers experienced occasional stockouts of some contraceptives (combined only contraceptives) which increased demand as there was now limited supply.

If you come to our site and see the numbers of people who are not eligible who would have come to get family planning services you will be shocked...I remember a policewoman came in her uniform and said I have come because I need family planning… She was pleading with me saying ‘she is a civil servant if you refuse to give me the family planning pills where do you expect me to get them from?’ But we couldn’t give her because she is above 24 years (Bulawayo, IDI06, Phase 2)

To minimize the chances of stockout, CHIEDZA providers started supplying one-month of oral contraceptives instead of the recommended three months’ supply.

Giving someone only a month’s supply was a challenge but we knew that a lot of people wanted the pills so we would offer a month’s supply instead of offering three months” (Bulawayo, IDI05, Phase 3)

Many young women now had to inconveniently return to CHIEDZA every month instead of every 3 months for oral contraceptive refills.

#### Covid-19 Affected Access to Family Planning

At the start of the COVID-19 pandemic, service delivery stopped for six weeks (Supplementary Table 1). According to the providers, young women who were due for their monthly contraceptive refills during this closure period, could not access CHIEDZA services.

I am thinking about those women who have their review dates drawing near in April and they would want to come but we are not there then what will happen? Because they are reliant on us to provide that service to them. (Bulawayo, IDI1, Phase 1)

When CHIEDZA reopened, providers reported that they experienced increased requests for pregnancy tests, and many of the women seeking services in that month were coming for family planning services, as they were not easily affordable in other places.

#### COVID-19 Affected Implementation Quality

Immediately after the intervention reopened, the workload increased for many of the CHIEDZA providers, due to increased volume of clients. While CHIEDZA staff strategized so that they could continue to provide health services for young people, they felt overworked, and exhausted.

On workload you would find that these days you go, and you might spend the whole day… and even to find food you would feel that you cannot go and eat leaving the booth just like that and the clients complaining that you are delaying us. There are some people who get annoyed with the waiting period but there is nothing we can do because there would be too many people. (Mashonaland East, IDI11 Phase 2)

The providers felt that the increases in workload jeopardized the service quality they could offer to young people:

So, the pressure on numbers I wouldn’t lie it was now making us divert from being a youth-friendly service because we were now chasing numbers. Because at the end we would even ask the youth workers at what number we are at and for real at the end of the day that became the main question. (Bulawayo, IDI02, Phase 2)

### Working with a Program Partner to Provide Commodities

This section describes the barriers and facilitators of working with another partner (PSZ) to provide short-term and long-acting family planning methods for young people in CHIEDZA. The trial’s pilot phase had established a need for LARCs and securing government supply of family planning methods was a challenge. Therefore, when the implementation period began (April 2019), CHIEDZA partnered with PSZ to offer both short-acting and long-acting family planning commodities during service hours (Supplementary Table 1).

We had a good working relationship [with PSZ]. They really assisted us because at first, before we were in partnership with the PSZ guys, we had clients who wanted long term but were disappointed because we could not offer it. (Bulawayo, IDI05, Phase 3)

However, PSZ was not able to consistently come to every CHIEDZA site during service delivery hours; and this left young women without ready access to implants and intra-uterine contraceptive devices**,** and services like implant removals.

Removals were the worst because there wasn’t any alternative unlike when someone says they want jadelle but later switch to another family planning that’s available at the site. For removals clients simply wanted it removed but still others did not have bus fare [to go to a non-CHIEDZA clinic] and we could not provide them with the funds. (Bulawayo, IDI03, Phase 3)

Implant removals were a challenge even when PSZ was present at CHIEDZA. CHIEDZA providers perceived that PSZ considered removals alone a misuse of already limited resources, especially when the target outcomes are implant insertion (uptake) and not necessarily removals. This constrained some CHIEDZA clients who only wanted implant removals.

The PSZ staff didn’t have kits and the packs to use so they would want someone who wanted to remove an implant and reinsert it because to them it wasn’t a target number or something like that…We had clients who wanted to get implants removed but they [PSZ] would tell them that they are not removing the implants on that day and the client should come back tomorrow. (Harare, IDI02, Phase 3)

Additionally, if clients wanted implant removals but did not get their implant inserted by PSZ, they were asked by PSZ nurses to return with proof of when, and by whom their implant was inserted, before getting the help they needed.

#### Family Planning Training-to-Practice

To mitigate against some the challenges being faced with the partnership model for providing LARCs, CHIEDZA nurses were registered to receive government-run training to be able to offer all methods, within CHIEDZA. This was a two-part training with a theory-based and a supervised practical component. The nurses attended theory sessions run by a parastatal government partner. They found the training to be beneficial in capacitating them to provide quality family planning services. However, completing the practical component of the training was not feasible. For the practical, a theory-trained nurse is required to insert ten implants and ten intra-uterine contraceptive devices under the supervision of designated personnel. CHIEDZA nurses could have done this practical with the supervision of qualified nurses. However, this was not possible as partners could not spare commodities to train colleagues during a national shortage.

I did not do all the procedures [IUCD] for me to qualify… for one to do these procedures they should at least have done ten procedures. With IUCD [intra-uterine contraceptive devices] I need supervision and about six procedures then I am good. I can do implants though. (Bulawayo, IDI05, Phase 3)

Although there was consensus that providing a range of family planning modalities within CHIEDZA was optimum, this was not possible because of the incomplete training.

#### Adapting the Partnership with Program Partner

As CHIEDZA providers could not complete LARC training, and PSZ could not always be present on all CHIEDZA days, the service delivery model for family planning was constantly adapted to ensure that as often as possible, young women had access to the full range of family planning methods (Supplementary Table 1). Instead of PSZ committing to come to all CHIEDZA sites, the young women at CHIEDZA would instead be referred to a PSZ site or clinic that was not at CHIEDZA for implants and intra-uterine contraceptive devices.

So now PSZ would come and if PSZ is not there we would refer them to a PSZ clinic so that they would get checked first if it’s hormonal imbalance or not and resort to using the loop (Harare, IDI02, Phase 3)

According to the providers, establishing, effectively implementing, and maintaining this adapted referral-based system was challenging. They perceived that this system diverged from CHIEDZA’s free, youth-friendly one-stop-shop integrated model, as young women would have to go to a non-CHIEDZA provider to access their contraceptive of choice. At non-CHIEDZA facilities young women were not prioritized over existing/other clients and sometimes these facilities did not have enough commodities and passed that cost onto young women.

So sometimes clients will need to bring their own sterile blades and at times the client won’t even have a dollar to buy the blade. it’s now the same as saying that the service is no longer free as compared to when they come on site to us. Now they have to incur transport costs and go to the PSZ centers in the city or a specific place that they are referred to. (Bulawayo, IDI02, Phase 2)

Between July–September 2020, due to organization-level changes and targets, PSZ was able to commit to bringing its services to the CHIEDZA sites again, so that young women wouldn’t have to go to another place for implants or intra-uterine contraceptive devices (Supplementary Table 1).

The lady [from PSZ] comes to every site we have... We first oriented her about CHIEDZA services and our client flow. PSZ services are also for free here, and there is no age limit for their services. So, a youth coming through even for PSZ services only, first passes through the youth and then they go through to the [health] booth. We talk to them and register them in our tablets. If they want an implant, we direct them to PSZ. If we are not busy one of us goes with them to PSZ so that we can also have the hands-on experience of doing implants. (Bulawayo, IDI03, Phase 2)

As before, it was not always feasible for PSZ to be present at the CHIEDZA community centers even when clients had been mobilized for LARC services. The CHIEDZA providers perceived that this was due to differences in ethos between CHIEDZA and PSZ. For them, PSZ was target-driven (uptake of contraceptives), whereas CHIEDZA was focused on youth-friendliness, such that the small numbers of young women at CHIEDZA who requested LARCs would be at the expense of PSZ’s targets.

When we got to our sites I remember in [Cluster 1] and [Cluster 3], we would get their clients already waiting for PSZ but then they would not show up…. we would ask them why they failed to come, and they would tell us that they went somewhere where a lot of clients turned up. We told them that you are losing trust of people who are in our clusters and want to access the service.... Their target were numbers and as CHIEDZA we didn’t give them the numbers at all (Harare, IDI02, Phase 3)

Therefore, the availability of implants or intra-uterine contraceptive devices ranged from site to site depending on if PSZ was present or not.

#### Maintaining Function but Shifting Implementation

In some instances, when the PSZ team was not able to come and offer LARCS, the CHIEDZA providers noted that they would pre-book CHIEDZA clients so that they could come and be served with LARCs on a day that PSZ would come for services:

Sometimes we would prebook clients and tell them to come on such a date that would have been set by the PSZ people. Pre-bookings were for [CHIEDZA cluster 1], [CHIEDZA cluster 2] and [CHIEDZA cluster 3] (Bulawayo, IDI05, Phase 3)

In other instances, the CHIEDZA providers would offer such clients the methods that were available at CHIEDZA. These were not always the client’s preferred choice, but clients took them up.

Not all our clients preferred short-term methods, and PSZ would disappoint us a lot of times. A lot of clients would ask for the long-term methods and say ‘if you give me the pills I will forget. I need a method that can stay for a very long time without remembering or forgetting’ (Harare, IDI10, Phase 3)

#### Feasibility of Offering Long-Acting Reversible Contraceptives

Some of the providers considered the service delivery model with PSZ to be a more suitable option for offering LARCs at CHIEDZA, compared to being fully trained and having to provider the LARCs within the CHIEDZA integrated care model. The CHIEDZA nurses felt that if they had to insert implants and intra-uterine contraceptive devices, it would compromise the quality of other HIV and SRH services as they would not have adequate time to do it all.

It would have been more work to insert long-term methods because you have to practice the inserting technique and setting up with packs involved. So, it was going to be added work for the nurse. That is why PSZ focuses specifically on inserting and removing LARCs only because you must watch out for infection and after every client you make sure that the place is clean to make sure that the place doesn’t get infections (Harare, IDI02, Phase3)

### Unintended Consequences of Intervention Adaptations

Responsive adaptations to the family planning intervention resulted in unanticipated effects that are presented in this section. When PSZ was at the sites, clients eligible for CHIEDZA but only needing PSZ services still had to follow the CHIEDZA client flow before engaging with PSZ services. In some instances, the researchers observed that young people spent all morning at the center waiting in line or in a long consultation alternating between the CHIEDZA and PSZ booths. In one instance, a client who wanted her implant removed waited all morning (~ 5 h), only to be asked to go home and return with proof of when and by whom her implant had been inserted.

## Discussion

Our study provides a practical illustration of the complexities of delivering a family planning intervention, and the adaptations made in response to these complexities. We conducted an exploratory qualitative process evaluation study that sought to assess fidelity, feasibility, and quality of implementing an integrated family planning service delivery model. Specifically, we looked at CHIEDZA provider experiences, and the response to contextual factors in the delivery of family planning services within CHIEDZA. Our study examined the contextual elements like the national shortage of family planning and the COVID-19 pandemic that disrupted fidelity and catalyzed adaptation. Quality family planning service delivery in CHIEDZA was envisioned as one that was free, offering both short and long-acting reversible methods, and delivered by youth-friendly, adequately trained providers.

Our study describes adaptations made to maintain this quality and the respective feasibility. Incomplete training for CHIEDZA providers led to a change in the delivery model where another organization was brought in to provide the full contraceptive method mix for young women. This had unintended consequences around youth-friendliness, wait times, and provision of long-acting reversible contraceptives.

Our study investigated the implementation outcomes: fidelity, quality, feasibility, and adaptations. Some of these outcomes straddle both process evaluations and implementation research frameworks (Quasdorf et al., 2021). Assigning theoretical allocations to these outcomes was not central to this study’s goal. Rather we sought to provide a demonstrative experience that was produced within a process evaluation setting and illustrates how implementation fidelity can be tracked, the viability of adaptations, and the impacts on feasibility and quality.

Our study demonstrated the implementation of a complex intervention (Fig. 1) within a complex set of partnerships or networks, and a dynamic and complicated context (Supplementary Table 1). CHIEDZA’s family planning intervention was designed to respond to emerging challenges (Moore et al., 2021). Designing the intervention this way shifted the focus from fidelity as implementing the original intervention, to having effective adaptations subsumed into measures of fidelity that ensure integrity (Cannata et al., 2021; Ghate, 2016; Lanham et al., 2013). For this study, intervention integrity was the delivery of quality family planning services for young women.

There have been opposing arguments about fidelity and adaptation, and in trial instances, there is often an assumption that the components of an intervention would be standardized, and fidelity to the standard is maintained across all intervention sites (Moore et al., 2015). In our case, unexpected disruptions like the commodity shortage and COVID-19 led to adaptations. In the former case, the supply of oral contraceptives given to clients was reduced, and in the latter, CHIEDZA was classified as an essential service so it would not be closed during severe lockdowns. Implementation research has begun to move away from qualifying fidelity/adaptation to examining the impacts of intervention adaptations (Kirk et al., 2020). For our intervention, adaptations were necessary, but feasibility remained a genuine challenge throughout implementation.

Our study supports that fidelity and adaptation are not in opposition. Implementation is itself a social process entangled in its context (Davidoff et al., 2008) such that the meaning of ‘*interventions as intended’* (fidelity) may differ for the various stakeholders who have to adopt it within the same context (Greenhalgh et al., 2004). Skilled implementers’ active attempts to make an intervention more suited to its population or setting, should not be considered poor fidelity (Bumbarger & Perkins, 2008), and can have an influence on users’ acceptability of the intervention. Our providers’ deviations from the original intervention design to responsively adapt, while remaining consistent to the theoretical and functional underpinnings of the intervention (Brand et al., 2019; Hill et al., 2020) may contribute to understanding interventions that are context-resilient in the long run. Therefore, there is a need to support and execute methods and evaluation designs that reflect the fluidity and often unpredictability of social contexts.

Attempts to examine how different adaptations may enhance (or not) the likelihood of interventions being transferable or scalable have remained ambiguous due to a dearth in clarity when conducting and reporting adaptations (Miller et al., 2020; Sundell et al., 2016). Our approach to responsive adaptations, and the learning from this approach, contributes to establishing and visualizing how ‘flexibility within fidelity’ (Kendall & Frank, 2018; Mignogna et al., 2018) can be dynamic while also systematically accommodating adaptations as integral parts of fidelity.

Implementing the adapted service delivery model in partnership with PSZ affected quality. Without PSZ, the CHIEDZA nurse would have had to dedicate significant time only inserting implants or Intra-uterine contraceptive devices, at the expense of other nurse tasks like sexually transmitted infections and antiretroviral therapy care. Having a partner organization available for insertions prevented the family planning intervention from potentially obstructing the CHIEDZA integration model overall. When PSZ was present, they were able to merge into the CHIEDZA model well enough and offer long-acting reversible contraceptives to CHIEDZA clients. On the other hand, their inability to provide commodities on every CHIEDZA service day and the target-driven nature of their work, diluted the intended quality components of CHIEDZA like short wait times, ready availability of full range of contraceptive methods, and youth-only spaces.

Process evaluations often take a retrospective approach (Webster et al., 2018). Our study’s strengths include conducting data collection and analysis of the process evaluation study during implementation of the intervention. This allowed us to capture real-time evolutions and dynamic processes of implementation, as well as notice opportunities to improve the intervention as it was being delivered. In studying systematic approaches to adaptations of interventions, qualitative research is useful to examine how the adaptations influence feasibility, acceptability, and intervention outcomes (Duggleby et al., 2020). Our study utilized a qualitative approach involving key stakeholders- the providers implementing the intervention (Moore et al., 2021) to guide and inform adaptations in the family intervention in CHIEDZA. Historically, process evaluation reports have not adequately elucidated context and its interplay with interventions (Greenhalgh et al., 2004; Hawe et al., 2004; Wells et al., 2012). As part of a process evaluation, our study demonstrates that what might be considered a failing of the intervention (challenges with LARC provision) highlights lessons for partnership approaches and adopting learning to actively respond to rather than ignore specific contextual conditions which shape implementation.

The limitations of the study are that we did not interview the providers from PSZ about their experiences implementing family planning within CHIEDZA. This would provide us with additional nuance about what works or doesn’t work for an intervention model like ours. Future studies could investigate implementation from the perspective of other stakeholders. Additionally, the main qualitative researcher (CM) was well-known to the CHIEDZA providers. While physical cues could not be noted during telephonic interviews, the existing and on-going relationship, and rapport between CM and the providers allowed for in-depth narratives. This established conducive rapport, may have increased the likelihood of courtesy bias as the providers became more familiar with her expectation regarding the process evaluation and implementation. This was mitigated by triangulation of different data sources and the presence of another researcher (RN) in the study.

## Conclusions

Context can be unpredictable such that implementation should be viewed as an emergent and dynamic process where responsive adaptations are necessary, and fidelity is not static. Anticipating that changes will occur is a necessary pre-condition of strong intellectual evaluation. This study was practical example of how process evaluation as a method of implementation science makes tracking responsive change vital. Tracking adaptations during a comprehensive process evaluation ensures lessons on feasibility of design, contextual factors and health system factors are responded to during implementation. Adaptations do not necessarily threaten implementation fidelity if the intended intervention is aligned to the function and not the form of the intervention. Rather, these adaptations should be tracked and considered as an integral process of delivering high quality services.

## Supplementary Information

Below is the link to the electronic supplementary material.

Supplementary file1 (DOCX 17 kb)

## Data Availability

The datasets generated and/or analyzed during the current study are not publicly available due the possible identification of participants, even after anonymization, but are available from the corresponding author on reasonable request.

## Abbreviations

CHIEDZA: Community-based Interventions to improve HIV outcomes in youth: a cluster randomised trial in Zimbabwe
CHW: Community health worker
IUCDs: Intra-uterine contraceptive devices
LARCs: Long-acting reversible contraceptives
PSZ: Population Services Zimbabwe
SRH: Sexual and reproductive health
