# Supplementary material for: Fidelity, Feasibility and Adaptation of a Family Planning Intervention for Young Women in Zimbabwe: Provider Perspectives and Experiences
Source: Glob Implement Res Appl. 2023 Mar 24;3(2):182–94. doi: 10.1007/s43477-023-00075-6 (PMC10037356; doi:10.1007/s43477-023-00075-6)
Supplement: Supplementary file 1 — Supplementary file1 (DOCX 17 kb) [file 43477_2023_75_MOESM1_ESM.docx]

Supplementary Table 1:Timeline of contextual factors and events interacting with family planning service implementation in CHIEDZA

| **Time point** | **Events** | **Context** | | **Adaptation** |
| --- | --- | --- | --- | --- |
| **Apr. 2019** | CHIEDZA began. Family planning commodities were meant to be provided by the government with CHIEDZA nurses offering oral contraceptives and injectables. | Reports of national shortage of contraceptive commodities at NatPharm (National Pharmacy) level.  CHIEDZA was not a registered health facility, therefore could not get contraceptive supplies through the MoHCC system. | | The PSZ partnership was established. PSZ staff would come to CHIEDZA sites and offer mixed- methods family planning products. |
| **May 2019** | PSZ attended CHIEDZA sporadically, to offer mainly implants and IUDs, and sometimes did not have enough commodities. If they had stock, they also offered oral contraceptives and Depo |  | | CHIEDZA decided to procure oral contraceptives and Depo and offer them inhouse. |
| **June 2019** | CHIEDZA nurses now offered oral contraceptives and Depo injectable, and uptake of family planning commodities increased. PSZ continued to attend CHIEDZA sporadically to offer LARCS. |  | | CHIEDZA clients who requested for LARCS in the absence of PSZ onsite, now got referral slips to go to a PSZ centre or clinic. |
| **July 2019** | PSZ stopped coming to CHIEDZA sites during July. | PSZ experienced challenges procuring enough family planning commodities and could not adequately support CHIEDZA | | LARCs are now completely provided through offsite referrals.  CHIEDZA nurses attend family planning training for implants to be able to offer LARCs inhouse at CHIEDZA |
| **Aug. 2019** | Young women were coming to CHIEDZA to access family planning commodities for their ineligible siblings, friends, and mothers. | In mid-August, CHIEDZA’s oral contraceptive supplier noted that there was no combined oral contraceptives in the country. | | CHIEDZA clients now get a health book recording when & type of contraceptives taken. They would need to bring this book with them for their next refill.  CHIEDZA would now offer 1 month supply of combined oral contraceptives instead of 3 months per national guidelines, to mitigate against shortage |
| **Sept. 2019** | CHIEDZA providers face challenges completing the practical training to be able to offer implants due to scarcity of family planning commodities. Commodities cannot be spared for training procedures. | Official announcement of national shortage in contraceptives. | |  |
| **Feb. 2020** | Ongoing national shortage of family planning commodities. | Implants are reported to be out of stock-nationally | |  |
| **Mar. 2020** | CHIEDZA nurses attend theory training for IUCD | COVID-19 is declared a pandemic | | CHIEDZA shut down on March 31^st^ |
| **May 2020** | Coordinators reported receiving calls and text messages on the CHIEDZA cell-lines from clients asking when CHIEDZA would reopen to access STI treatments, family planning and condoms.  CHIEDZA reopened on May 14^th^ as an essential service, observing all COVID-19 infection and prevention control measures. |  | | In addition to complying to national lockdown measures, CHIEDZA covid-19 adaptations *(published elsewhere)* included removal of social activities, installing handwashing, and sanitising stations, mandating social-distancing and ‘no mask no services’, moving the health booths outside for better ventilation |
| **June 2020** | High volume of clients after CHIEDZA reopened increased workload for providers. Providers noted decrease in the quality of service provision as they tried to serve many clients within a short period of time. | Lockdown measures included curfews that reduced the work hours for CHIEDZA providers.  June 19^th^, doctors, and nurses in Zimbabwe went on strike. | |  |
| **July 2020** | Requests for emergency contraceptive and pregnancy test increased. Young women noted having unprotected sex and running out of their contraceptive supply in the lockdown.  Young people were access family planning to resale.  In CHIEDZA communities, the clinics were referring youth to CHIEDZA for family planning and HIV testing, instead of serving them at the clinic | The national shortage and resultant exorbitant price of contraceptive commodities deepened and community members are complaining about it (Bulawayo) | | In mid-July2020, Bulawayo re-established partnership with PSZ. PSZ would come offer family planning at the CHIEDZA centres it was able to. For those centres where PSZ could not come, referrals to PSZ clinics/centres for LARCS would continue |
| **Aug. 2020** |  | mid-August, lockdown measures were eased, and work hours increased for the CHIEDZA teams | |  |
| **Sep. 2020-Mar. 2022**  **(Final Adaptation)** | **Harare and Mashonaland East** re-established relationship with PSZ. The initially consensus was that PSZ would come offer family planning at all the CHIEDZA centres, except for a few exceptions, where the PSZ teams already had prior commitments to other communities. In the latter case, PSZ would proactively inform CHIEDZA of their availability so that at the centres where PSZ does not come, the clients who want LARCS continue to be referred to PSZ clinics/ centres.  In practice, PSZ came to offer family planning (mostly implants and IUCDs) at the CHIEDZA centres it was able to. | | | |
| **Oct. 2020** | PSZ nurse in Bulawayo has not been coming to CHIEDZA all month | | PSZ faces resource challenges in trying to support CHIEDZA.  Oral contraceptives in short supply in Bulawayo. |  |
| **Dec. 2020** | Giving clients 1 month supply of oral contraceptives is considered to be driving client traffic as clients are coming back every month just for a refill.  CHIEDZA closes on December 18^th^ for the holidays. | | Clients continuing to come to CHIEDZA every month only for contraceptive refills is considered high risk behaviour for COVID-19 |  |
| **Jan. 2021** | CHIEDZA reopens as an established essential service. Family planning refills account for most of the client flow since CHIEDZA has closed for the holidays. | | A level 4 lockdown is announced due to increase in COVID-19 cases. | CHIEDZA returns to providing clients with 3 months’ supply of oral contraceptives on 18 January 2021. |
| **June 2021** | Reports that several clients who come to CHIEDZA to have their implants removed by PSZ have been turned away.  A recognized need to have CHIEDZA providers trained and able to offer LARCS independent of PSZ. | | PSZ functions in a specific manner for implant removals. If the client’s implant was not inserted by PSZ, the client often faced challenges the implant removed by PSZ. |  |
